# Research on Grain Refinement Mechanism of 6061 Aluminum Alloy Processed by Combined SPD Methods of ECAP and MAC

**DOI:** 10.3390/ma11071246

**Published:** 2018-07-20

**Authors:** Zhenwei Zhang, Junli Wang, Qinglong Zhang, Supeng Zhang, Qingnan Shi, Huarong Qi

**Affiliations:** 1 Institute of Material and Science Engineering, Kunming University of Science and Technology, Kunming 650093, China; zhangzhenweisamuel@163.com (Z.Z.); zhangqinglong58@163.com (Q.Z.); zhangsupeng07@163.com (S.Z.); shikust@vip.163.com (Q.S.); qihuarong@163.com (H.Q.); 2 Institute of Material and Science Engineering, Nanjing University of Science and Technology, Nanjing 210094, China; 3 Research Center for Analysis and Measurement, Kunming University of Science and Technology, Kunming 650093, China

**Keywords:** grain refinement mechanism, 6061 aluminum alloys, CECC, ECAP, MAC

## Abstract

Equal channel angular pressing (ECAP) and multi-axial compression deformation (MAC) are severe plastic deformation (SPD) processes that produce bulk nanostructured materials with ultrafine grains. The grains could be observably refined by multi-pass of ECAP and MAC. This research proposed new routes of cyclic equal channel compression (CECC), which combines ECAP and MAC to increase the mechanical properties of 6061 aluminum alloy. The tests, which are conducted through electron backscattered diffraction (EBSD) and transmission electron microscope (TEM), were performed on the grain size, recrystallization distribution, misorientation distributions, dislocations, and secondary phase distributions of CECC-processed 6061 aluminum alloys on the purpose of exploring the mechanism of grain refinement. MEM is the short form for the CECC processing route of MAC + ECAP + MAC, which is one ECAP pass between two MAC passes. The tests results showed that the average grain size could reach to as much as 1.1 μm after two MEM deformation circles named MEM-MEM, with the non-annealing average grain size being 21 μm and recrystallization annealed average grain size being 28 μm. The dislocation cells, which could be transformed into sub-grains with the increase of the strain, were formed by the slip and the accumulation of dislocations. The secondary phase was Mg_2_Si, which could prevent the refined grains from growing up again by pinning at the grain boundaries. Above all, the dislocation proliferation and secondary phases will both lead to the grain refinement.

## 1. Introduction

The mechanical properties of metallic materials could be enhanced through solid solution, work hardening, and grain refinement. The very fine-grained material processed by severe plastic deformation (SPD) methods shows better mechanical responses and improved physical properties. Among the different SPD methods, Equal-Channel Angular Pressing (ECAP) and Muti-Axial Compressing (MAC), which offer the opportunity to scale up the process to an industrial level [1,2], are both cost-efficient methods of imposing extremely large plastic strain to bulk materials with exceptionally small grain size. Cyclic equal channel compression (CECC) is a creative SPD method, which combines ECAP and MAC randomly so it can refine grains more effectively. In the ECAP process, the extrusion punch presses the sample into the die at a constant speed. The deformed dimensions, which could repeat the process for several times to accumulate large plastic strain [3], are identical to the initial ones. The sample produces uniform shear deformation at the corner of the die. After ECAP, the sample is compressed in the MAC mold where the cross section of the sample has the same size of the longitudinal section [4]. During MAC process, the sample is continuously compressed and elongated with the external load. The sample is rotated 90° and put into the mold for next circular compression after one pass of MAC for the accumulation of strains in all directions. The major advantage of ECAP and MAC processes is their abilities to produce large quantities of ultra-fined grains (UFGs) [5]. Several passes of ECAP and MAC significantly refine the grains and increase the strength of the material without changing the sample dimensions.

**Figure 1:**
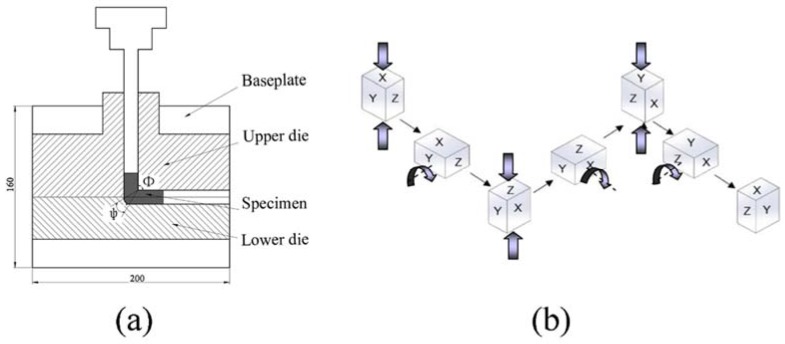
The diagram of 6061 aluminum alloy processed by CECC. (**a**) ECAP (Equal channel angular pressing); (**b**) MAC (Muti-axial compression) processes.

The intersecting channel angle and the corner curvature angle of the ECAP die are referred to as Φ and Ψ, respectively, in Figure 1a. In theory, the smaller the extrusion angle, the higher strain and the more significant shear deformation can be achieved per pass. However, if the angle Ψ is close to 0°, the hard deformation zone will be formed on the outside surface of the sample. The hard deformation zone will be evitable if the angle Ψ is greater than 20°. According to the finite element stimulation reported by Pucun Bai [6], the most practical angles in ECAP processing of aluminum alloys are Φ = 90° and Ψ = 30°. With these angles, the strain will be 0.962 for a single pass.

Many experiments were performed on the effect of process temperature during ECAP on the microstructure development of Al alloys. Goloborodko A and Weiyi Wang [7,8] believed that the room temperature during ECAP could result in the formation of finer equiaxed ultrafine grain structure. The sample is subjected to shear deformation at the corner of the two channels when passing through the die [9]. The results show that, under the cryogenic temperature, the suppress grain dynamic recovery can occur in the ECAP process, which could significantly refine the grain of aluminum alloy to the micrometer scale or even the nanometer scale [10]. Furthermore, Langdon et al. [11] reported that the mechanism of UFG materials obtained through MAC is that different shear zones constituted by many homogeneous shear layers intersect and react together, so they can increase the density of dislocations. As predicted, the material exhibited high work hardening rates in the final monotonic compression step. The deformation strain (ε) of 6061 aluminum alloys processed by MAC could be calculated through the following equation [12]:

(1) $$\varepsilon =\frac{\sqrt{3}n}{2}\mathrm{In}(\frac{H}{h})$$

where n, H, and h represent the deformation pass, the height before deformation, and the height after deformation, respectively.

The ultra-high strength and heat treatment commercial 6061 aluminum alloy Al-Mg-Si series is extensively used in the aerospace and construction industries as they possess high strength to density ratios as well as good corrosion resistance [13]. However, the commercial 6061 aluminum alloy did not achieve good strength and plasticity. Several passes of CECC could improve the mechanical properties of 6061 aluminum alloy.

The newly designed CECC routes include one MAC pass followed by two ECAP passes (MEE, which is the short form for MAC+ECAP+MAC) and one ECAP pass between two MAC passes (MEM). It is obvious that ECAP can lead to stress concentration and the preferred orientation of the grain, while MAC can disorganize this preferred orientation. The combination of ECAP and MAC, which could produce UFG materials in less passes, contributes to the achievements of better extrusion effect. Therefore, it has practical significance for the study of the mechanical properties, microstructure evolution, and grain refinement mechanism of aluminum alloys processed by CECC. The single ECAP and MAC deformations were conducted to make the comparison with CECC. The aim of this work is to explore the best CECC route and research the grain refinement mechanism of 6061 aluminum alloy processed by CECC. The first stage of work includes the extrusion of samples processed by MEM, MAC, 3-pass of ECAP, and 3-pass of MAC. The second stage of work includes the study of the microstructure evolution and phase transformation by electron backscattered diffraction (EBSD) and transmission electron microscope (TEM), both of which are from Eindhoven, Netherlands, manufactured by FEI. 

## 2. Materials and Methods

Commercial 6061 aluminum alloys (Al-Mg-Si alloy) manufactured by Southwest Aluminum Co., Ltd. (Chongqing, China) were supplied in the as-cast condition, the chemical composition of which is provided in Table 1. All samples were subjected to recrystallization annealing under the same conditions (350 °C for 2 h, heat for 1 h and thermal insulation for 1 h). The annealed samples (10 × 10 × 20 mm^3^) were processed in the mold of ECAP and MAC, with the long dimension being vertical to the mold. The intersecting channel angle Φ and the corner curvature angle Ψ of the ECAP die were 90° and 30°, respectively. The samples were presses up via route B_C_ (90-degree rotation clockwise after each pass), which is the most efficient processing route for grain refinement reported by many researchers [14,15]. In this research, ECAP and MAC were combined by different methods to further improve the mechanical properties of 6061 aluminum alloy. Figure 1 shows the diagram of 6061 aluminum alloy processed by CECC. The CECC experiments were carried out at room temperature, in order to prevent significant grain growth of the new grains.

The experiments and mechanical properties tests were performed in the SHIMADZU tensile testing machine. Vickers microhardness values (HV) were recorded on a cross-section plane. A total load of 100 gf was imposed and the dwell time was 15 s for each sample. The compression die and specimen interface were lubricated by molybdenum disulfide paste in order to lower the friction between the billet and the die. When taking specimens, 6061 aluminum alloys can be taken out by separating the upper die and lower die in the ECAP process. In the MAC process, Al-6061 alloy can be taken out in the sampling space when the die holder was taken out before extruding the sample on the tensile testing machine.

The Transmission Electron Microscope (TEM, manufactured by FEI, Eindhoven, Netherlands) test was performed in the Tecnai G2 TF30 S-Twin. The dislocations and secondary phases could be analyzed by TEM. When preparing TEM samples, samples with dimensions of 10 × 10 mm^2^ and thickness of 2 mm were cut from billets perpendicular to the processing direction. The thickness of samples was less than 100 μm after being mechanically thinned. Afterwards, 3 mm diameter discs were punched and the samples were then chemically etched in 10% solution of HClO_4_.

The grain size was measured by intercept methods. An intercept is the segment of the teat line that overlays one grains when counting the intercepts. Then, make counts on three to five blindly selected and widely separate files to obtain a reasonable average for the specimen. The uniformly distributed test lines are used for an independent for equiaxed structure. Finally, at least 300 grains need to be count for an approximation estimate of a sample. 

The microstructure of the samples was investigated by Nova Nano SEM450 field-emission scanning electron microscope (FE-SEM, manufactured by FEI, Eindhoven, Netherlands) equipped with an Electron Backscatter Diffraction (EBSD) camera. The grain misorientation distributions and recrystallization distributions of 6061 aluminum alloys processed by different routes of CECC could be analyzed by EBSD. When preparing EBSD samples, specimens are mechanically polished on the polishing machine with Al_2_O_3_ suspension liquid and the particle size of less than 0.1 μm. After that, the sample was electropolished in the solution of 10% perchloric acid and 90% ethyl alcohol for about 40 s, followed by the iron etching for 2 h with the electricity of 15 μA to make sure that there were not any scratches and stress on the surface of specimens. The etching conditions were selected according to the previous studies [16]. The HKL CHANNELS software was used to performed EBSD data visualization and post processing.

The creative CECC deformation routes include one MAC pass followed by two ECAP passes (MAC + ECAP + ECAP, MEE) and one ECAP pass between two MAC passes (MAC + ECAP + MAC, MEM). Three passes of continuous equal channel angular pressing (3-pass of ECAP) and three passes of continuous muti-axial compression (3-pass of MAC), which were compared with MEM and MEE, were also conducted. The samples processed by MEM, MEE, and 3-pass of ECAP and MAC were chosen for the EBSD detections and the samples processed by MEM and MEE were chosen for the TEM detections. Moreover, MEM-ed and MEE-ed samples were extruded in another CECC deformation circle—the double MEM and MEE processes named MEM-MEM and MEE-MEE, which were equivalent to six passes of CECC in total so it can achieve more grains in nanoscale and improve the mechanical properties of 6061 aluminum alloys. The samples processed by MEM-MEM and MEE-MEE were also chosen for the TEM detections. These steps were taken to better evaluate the effect of grain size and texture, thereby demonstrating the influence of any deleterious secondary phases interacting with the dislocations.

## 3. Experimental Results 

### 3.1. Mechanical Properties

Tensile tests were conducted by using samples with the dimension of 10 mm in width and 20 mm in length. Table 2 illustrates the results of tensile tests. In fact, the results of tensile test are consistent with the results of microhardness test. The ultimate strength of the sample processed by MEM reached 320 MPa, which was 2.5 times of the undeformed and annealed one and was the highest among four SPD methods. The elongations of the sample processed by 3-pass of ECAP, 3-pass of MAC, MEM, and MEE are 10.9%, 10.2%, 12.8%, and 11.1% respectively, which were a bit lower than the undeformed and annealed sample. The microhardness of the sample processed by MEM which reached 115.2 Hv was also higher than 3-pass of ECAP and MAC, which reached 92.5 Hv and 88.1 Hv respectively. In conclusion, the tensile strength and microhardness of 6061 aluminum alloy processed by CECC increased more sharply than 3-pass of ECAP and MAC and the specimen processed by MEM had the best mechanical properties.

### 3.2. Grain Size

**Figure 2:**
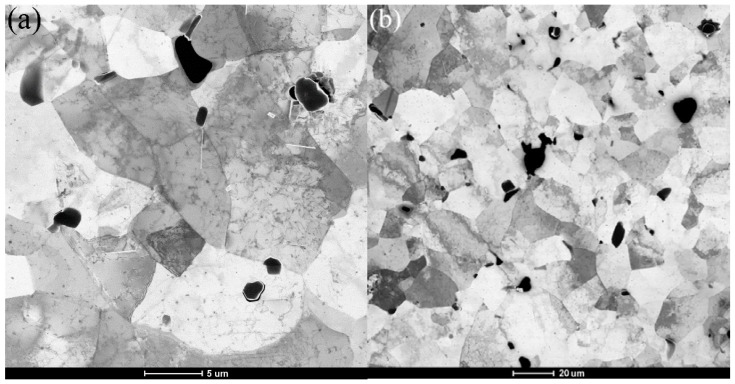
The Transmission Electron Microscope images of homogenized (before CECC process) 6061 aluminum alloy observed with different magnification. (**a**) 5 (μm); (**b**) 20 (μm).

**Figure 3:**
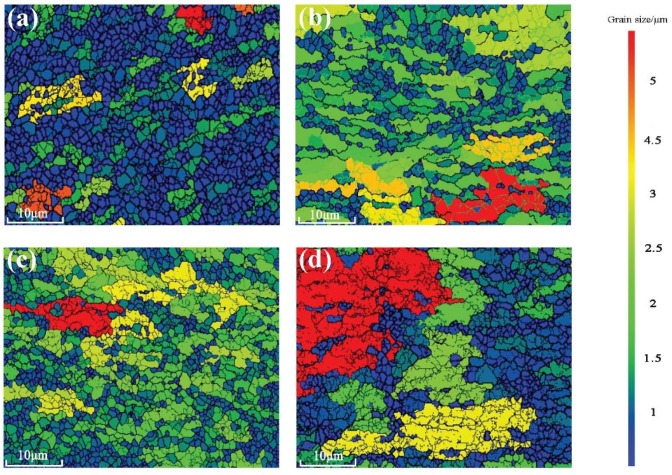
The EBSD microstructure of aluminum alloy processed by different routes of CECC. (**a**) MEM; (**b**) MEE; (**c**) 3-pass of ECAP; (**d**) 3-pass of MAC.

The grains became finer after the SPD process and would generate more grain boundaries [17]. The grain size distribution is calculated under the assumption that the minimum misorientation of grain boundary was 15°. The strength and the toughness of the material could be improved by the grain refinement [18]. Figure 2 displays the TEM diagrams of recrystallization annealed and undeformed 6061 aluminum alloys. The secondary phases of 6061 aluminum alloy were very few and the morphology of grains were not very equiaxed. Figure 3 displays the EBSD microstructure of aluminum alloy processed by different routes of CECC. The different colors represent different grain size in the diagrams. The dark blue indicates the smallest grains and the red represents the largest grains. The step size was 0.1 μm, the voltage was 20 kV, and the grid was 421 × 313. The Al particles sizes are significantly smaller, which indicates that the grain size of the sample was effectively refined after the CECC. However, after MEE deformation, as shown in Figure 3b, a small amount of coarse grains appeared in fine matrix because some part of the specimen was not fully deformed during ECAP deformation.

The average grain sizes of 6061 aluminum alloys processed by MEM, MEE, 3-pass of ECAP and 3-pass of MAC are 1.2 μm, 1.6 μm, 2.1 μm and 2.8 μm, respectively according to the Table 3. The grains of the sample processed by MEM and MEE were much finer and more equiaxed than 3-pass of ECAP and MAC. Although some larger grains also existed after the deformation, most of grains had experienced significant refinement and became homogenized.

### 3.3. Misorientation Distributions

**Figure 4:**
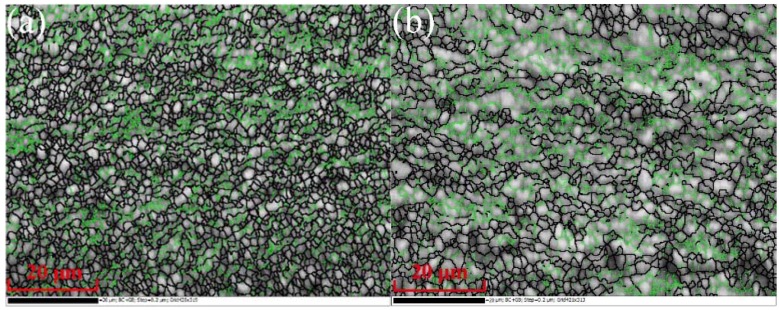

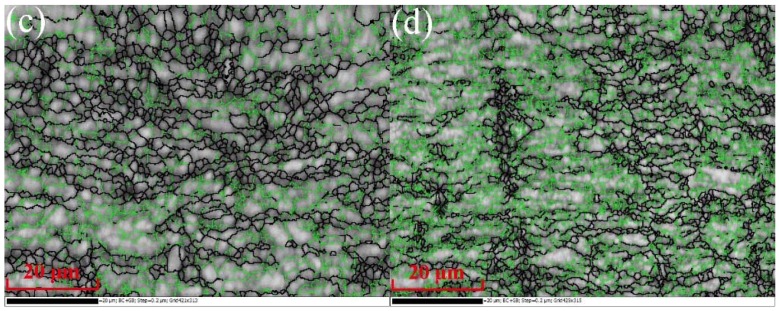
Grain misorientation distributions of 6061 aluminum alloy. (**a**) MEM; (**b**) MEE; (**c**) 3-pass of ECAP; (**d**) 3-pass of MAC.

Based on the function of orientation measurement of EBSD and analysis software, datum of the misorientation distributions between the neighboring pixels and the types of grain boundaries could be acquired. Figure 4 displays the grain misorientation distributions of 6061 aluminum alloys after MEM, MEE, 3-pass of ECAP, and 3-pass of MAC. The green area represents the low-angle grain boundary and the black area represents the high-angle grain boundary. The results show the distribution of grain boundary misorientations. The grain boundaries were less sharp and, in many cases, very blurry as shown in Figure 4. After 3-pass of ECAP and MAC, the material was already fragmented. Some small areas that were not divided into smaller grains were also visible. However, during the MEM process, the grain size of Al-6061 alloy was more uniform and homogeneous. The alloy generates a large number of equiaxed grains because of the static recrystallization. The low-angle grain boundaries of 6061 aluminum alloys processed by MEM and MEE have occupied a small proportion. The present results show that SPD provided new ways for grain boundary design through the fabrication of UFG and nanocrystalline metals [19]. The structure of new grains boundaries that have formed after MEM and MEE processes had a significant effect on the mechanical properties of UFG materials.

**Figure 5:**
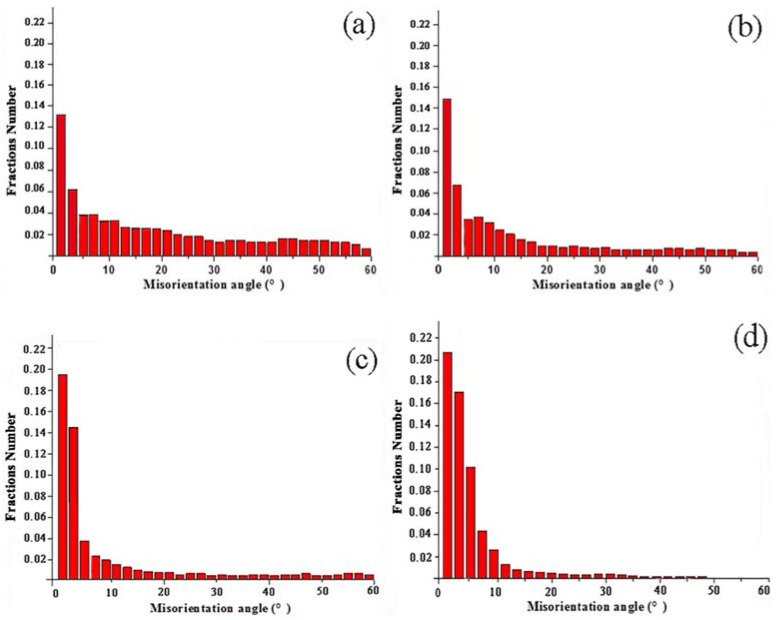
The grain misorientation angles of 6061 aluminum alloys. (**a**) MEM; (**b**) MEE; (**c**) 3-pass of ECAP; (**d**) 3-pass of MAC.

The EBSD images obtained under various conditions of electric current treatment were further analyzed for misorientation angles. The plastic deformation caused by high stress is the reason for the formation of dislocations in 6061 aluminum alloys. Figure 5 displays the histograms that represent the average misorientation angles of 6061 aluminum alloys for each processing condition. The high misorientation angles of specimens processed by MEM and MEE were more than ECAP and MAC process. The average misorientation angles of MEM were around 10°~15° because the dislocation moved toward the sub-boundary, forming the UFG structure with high-angle boundaries. Moreover, the coarse grain was broken in the beginning of the deformation, with large number of dislocation tangles inside the grain. As a result, the dislocation proliferation produces low-angle grain boundaries, so the amount of low-angle grain boundaries (LAGBs) were more than these of high-angle boundaries (HAGBs). However, when the CECC deformation continues, the LAGBs will keep reducing and the HAGBs will demonstrate homogeneous distribution. Therefore, the number of grain boundaries will be increased and the grains will be significantly refined.

### 3.4. Recrystallization

The recrystallization process of aluminum and aluminum alloys is the process of generating the new nuclei on the deformed substrate. The grain orientations spread inside the grains were significantly reduced, and the grains became more homogeneous. The characteristic band structure after deformation disappeared, and the grains became more equiaxed. The crystal grain size of cold deformation metals became relatively small after the complication of recrystallization. Relying on the migration of large angular grain boundary, the core of recrystallization growth can eliminate and change the original deformation texture. The grain size would become smaller if the intense shape variation occurred. Recrystallization is one of the most important processes for the elimination of the defects in deformed metals and alloys and therefore the modification of their microstructure and properties [20].

**Figure 6:**
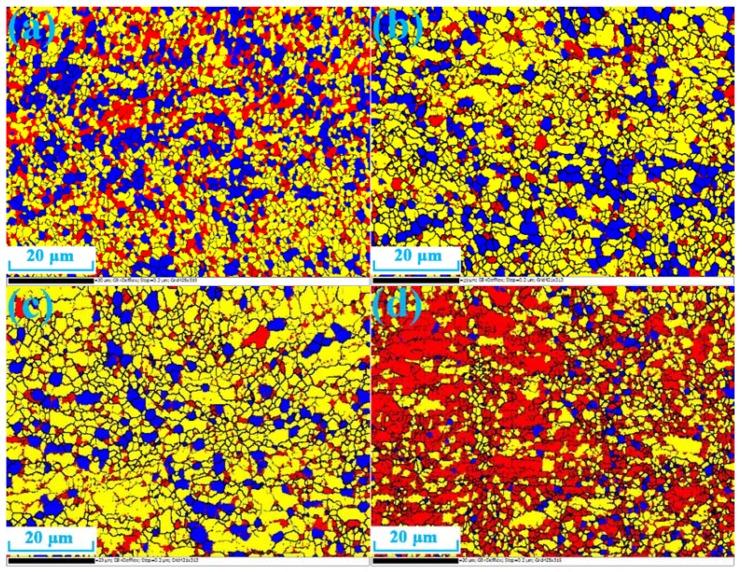
Recrystallization distribution of 6061 aluminum alloys processed by different routes of CECC. (**a**) MEM; (**b**) MEE; (**c**) 3-pass of ECAP; (**d**) 3-pass of MAC.

Figure 6 shows the recrystallization distribution of 6061 aluminum alloys, and Table 4 shows the corresponding statistic datum. The blue region represents the recrystallized grains which are equiaxed and approximately circular and the red region represents the deformed grains which have a tendency to elongate in one direction. The sub-structured grains with no significant orientation are represented by the yellow region. The dislocation density is much higher because dislocations are entangled inside the sub-structured grain. The results show that when the sample is processed through the MAC process, the number of the deformed grains is the largest; when through the MEM process, the number is the second largest; when through the MAC process, the number of sub-structured grains was the smallest, and that the recrystallized grains of the MEM process occupied the most area. Through the result, we can conclude that the recrystallized grains which weakened the preferred orientation of crystals were easy to be formed after the MEM process. The sub-structured grains were unstable, which could be transformed into the recrystallized grains easily after the MEM process.

## 4. Microstructure Observations

**Figure 7:**
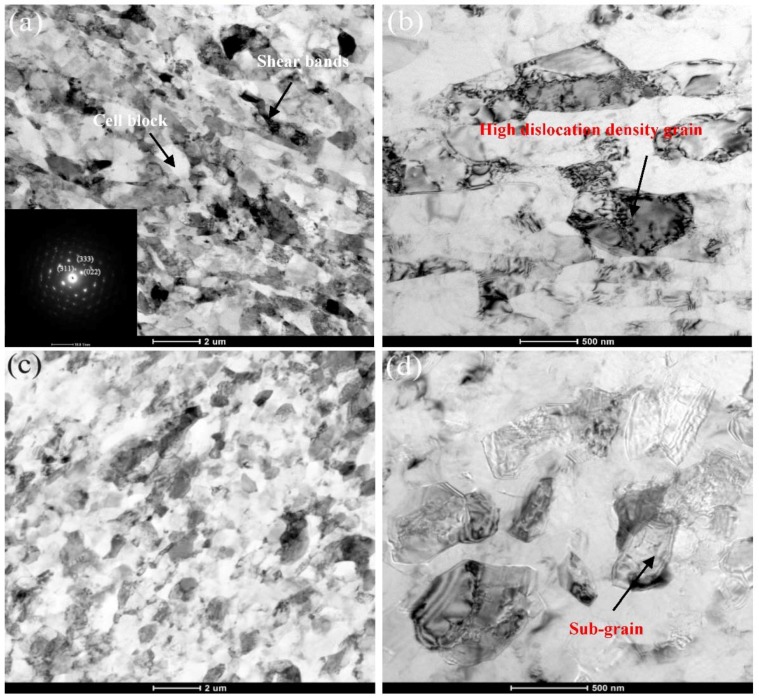
The TEM images showing general microstructure of Al-6061 alloy in the deformed stated processed by MEM and MEE. (**a**,**b**) Al-6061 alloy processed by MEM; (**c**,**d**) Al-6061 alloy processed by MEE.

Plenty of researchers such as Wronski S, Renguo G [21,22], and so on have discussed the mechanism of grain refinement in aluminum alloys processed by ECAP. It was shown that grain refinement is caused by dynamic recrystallization and the new grains are formed, which lead to the homogenized distribution of finer grains. The electron microscopy analysis was conducted to carry out a precise investigation of the grain refining mechanism of 6061 aluminum alloys processed by CECC. Figure 7 shows the TEM bright field images of the microstructure of Al-6061 alloy in the deformed state processed by MEM and MEE. CECC processes of MEM and MEE lead to a significant cell and grain size reduction to sub-micrometer scale to 1.2 μm and 1.6 μm, respectively, which were measured with the line intercept method. The grain refinement effect of MEM deformation was much better than that of MEE deformation.

In the most intensively deformed zone, finer grains were generated with the effect of extremely shearing force. Isometric crystals with high-angle grain boundaries were increased and elongated in the longitudinal section because of the interaction of shearing force, so the long strip shear bands could be observed in Figure 7a, which was the MEM processing. The slip bands with the shape of the stripe patterns were generated by the high density of dislocations. Figure 7a also reveals the results of the corresponding selected area electron diffraction (SAED) patterns, in which precipitates are distributed homogeneously within the matrix. It is obvious that the GP zones predominantly populate the grain interior. Besides, the dislocation mechanism added azimuthal misorientation to the SAED pattern reflections and formed fine sub-grain structures [23]. In addition to the diffraction spots from the aluminum substrate, the other set of diffraction spots appear. In conclusions, the precipitates in the MEM-ed 6061 aluminum alloy were mainly GP zones and the secondary phase was Mg_2_Si as verified by the SAED pattern.

The regions containing a high density of dislocations and sub-boundaries were marked in the Figure 7b. Figure 7b shows a lot of dense tangled dislocations remained inside the cells and grains, which result in an increase of strain accumulation in the material after CECC process. The tangled dislocations, which were clearly visible within the grains and tangled with large precipitates adjacent to the grain boundaries, were formed through the deposition. The dislocation tangle zone with irregular shape, which could be observed in the grain interiors, was stacked together to form a dense dislocation wall. The dislocation density of the grains was increased after MEM and MEE deformation while LAGBs are transformed into HAGBs, which is consistent with the EBSD results. The increase of the HAGBs would lead to the increase of the ductility and strength of 6061 aluminum alloys and the improvement of the microstructure homogeneity. Some sub-grains formed by the union of grain internal dislocations could also be observed in Figure 7b. The dislocation cells were formed by the slip and the accumulation of dislocations, which could be transformed into sub-grains with the increase of the strain. The dislocation density of sub-grains was relatively low. Moreover, Ciemiorek M [24] found that the accumulative action of the strengthening mechanisms caused by irregular dislocation tangles and the organized substructures resulted in a significant enhancement of the strength.

Figure 7c,d shows the typical microstructure of the MEE process. The deformed grains have a tendency to be elongated. The elongated grains could be broken into equiaxed grains, which cannot form inside the grains but along the grain boundaries. The process of MEE deformation does restrain dislocation movements inside grains and cells, contributing to some extent to the suppression of the cell recombination to form the grain boundary. In conclusions, the effect of grain refinement is much more remarkable after CECC deformation. The dislocation cells were formed by the slip and the accumulation of dislocations, which could be transformed into sub-grains with the increase of the strain.

**Figure 8:**
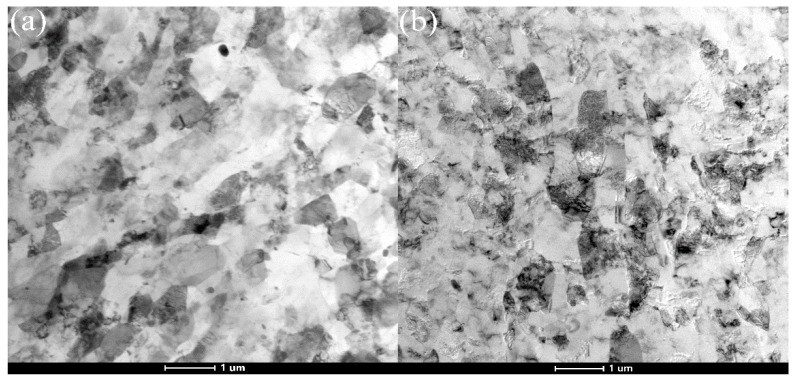
The TEM images showing general microstructure of Al-6061 alloy in the deformed stated processed by MEM-MEM and MEE-MEE. (**a**) MEM-MEM; (**b**) MEE-MEE.

The dislocations accumulate and annihilate at the sub-grain boundaries, which make the sub-grain boundaries gradually transformed into grain boundaries. Samples were extruded by MEM-MEM and MEE-MEE for the reason that it can reduce the sub-grains and substructures observed in the 6061 aluminum alloys processed by MEM and MEE. The TEM results were shown in Figure 8. The average grain sizes of Al-6061 alloys processed by MEM-MEM and MEE-MEE were 1.1 μm and 1.4 μm, respectively, according to Figure 8. The grain refinement effect of MEM deformation was much better than that of MEE deformation. The low-angle grain boundaries decreased while the high-angle grain boundaries increased, which consumed a lot of dislocations. Besides, the dislocation density in subgrains decreases significantly. Much more new grains without dislocation tangle, which has great refinement effect to the original grains, could be observed in Figure 8a after MEM deformation. The grain refinement effect of MEM deformation was much better than that of MEE deformation.

There are many secondary phases in Al-6061 alloy which are not scattered homogeneously. Meanwhile, 6061 aluminum alloys would be subjected to the strong shear deformation in all directions which leads to the homogenization of the force of the secondary phases in the alloy after MEM-MEM and MEE-MEE deformation. Some fine and uniform distributed secondary phases were precipitated on the aluminum matrix. It was reported by M. Cabibbo [25] that the process of ECAP could induce secondary phase precipitation sequence along the newly formed cell boundaries in heat-treatment alloys such as the AA6000-series. Hong Yu pointed out that the slipping dislocations interlace with each other and are blocked at the interface of hard brittle particles and α-Al matrix, leading to a greater shear force on the brittle particles [26]. In this study, the Al-6061 alloy has the tendency to initiate the precipitations of secondary phases. The precipitates in the microstructure consist of larger secondary phases mostly distributed along the grain boundaries and finer secondary phases distributed evenly within the grains [27]. With the increase of extrusion passes, the secondary phases were gradually refined.

**Figure 9:**
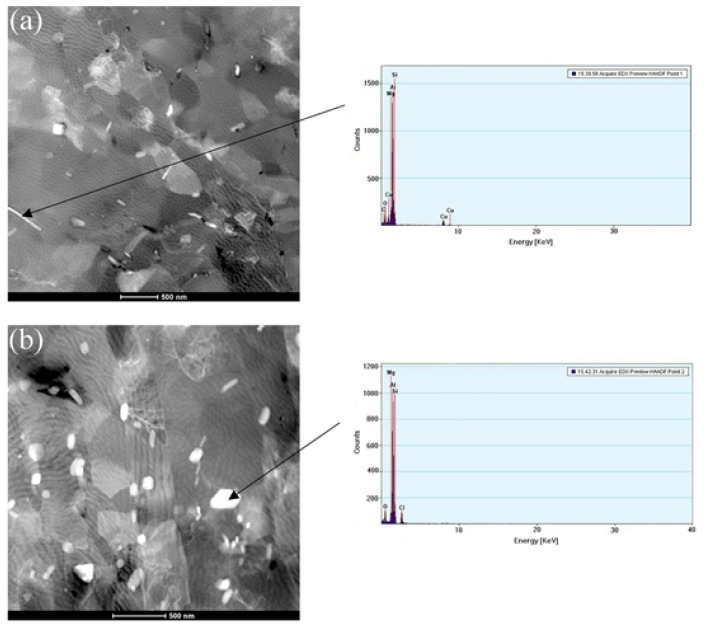
The TEM images showing the secondary phase of 6061 aluminum alloy in the deformed state processed by MEM and MEM-MEM and the corresponding EDS spectra of the elementary composition of secondary phase. (**a**) MEM; (**b**) MEM-MEM.

Figure 9 displays the evolution of secondary phases processed by MEM and MEM-MEM. The shape of the secondary phase was changed from the long needle to the short rod. After MEM deformation, large plate-like secondary phase of Mg_2_Si in 6061 aluminum alloys fragmented to various extents and the average size of Mg_2_Si particles was reduced to around 400 nm, which exhibited remarkable refinement properties. With the decrease in the average size of Mg_2_Si particles, the average grain size of 6061 aluminum alloy decreased. Moreover, the enhancement in size reduction and distribution homogeneity of the Mg_2_Si was more prominent with increasing CECC passes.

The secondary phase was further deformed into thinner and longer flakes in the MEM-ed sample and were distributed homogeneously after MEM-MEM deformation. At the same time, it was found that the secondary phases had the pinning effect on the grain boundary, which may hinder the grain growth and would promote the grain refinement in consequently. Figure 9 also shows the elementary composition of secondary phases of Al-6061 alloys observed by energy spectrum analysis. According to Figure 9a, the spectrum peak of Al was the highest. However, C and O were impurity elements. The spectrum peaks of Mg, Si and Cu were obvious, while other elements were hardly seen in the spectrum. Moreover, as is shown in Figure 9b, the spectrum peaks of Al, Mg and Si were also much more obvious. The impurity element of Cl was derived from HClO_4_. Therefore, it can be deduced that the main secondary phase is Mg_2_Si [28,29]. It was also reported that there might be CuAl_2_, β-FeSiAl_3_ phases in A1-6061 alloy [30], but their spectrum peaks were hardly to be seen due to their low contents. The secondary phase of Mg_2_Si function as nucleation substrate, and the refining performance of 6061 aluminum alloy. Moreover, the secondary phase was pinned at the grain boundaries, preventing the refined grains of growing up again. In conclusion, the secondary phases will promote the grain refinement during CECC process.

## 5. Conclusions

The grains of 6061 aluminum alloy processed by MEE, MEM, and 3-pass of ECAP and MAC were significantly refined. The mechanical properties of 6061 aluminum alloy have been significantly improved. The newly-designed CECC routes have refined grains more effectively to the sub-micron scale than 3-pass of ECAP and MAC. The grains have refined from 21 μm before annealing and 28 μm after recrystallization annealing without deformation to 1.1 μm after MEM-MEM deformation. The grain refinement effect of MEM deformation was much better than that of MEE deformation. The specimens processed by MEM, MEE, and 3-pass of ECAP and MEM were analyzed through EBSD, respectively, and the conclusions are made below.

1. During the MEM process, the grain size of Al-6061 alloy was more uniform and homogeneous. A lot of equiaxed grains of 6061 aluminum alloys were generated because of the static recrystallization.
2. The MEM-processed specimens mainly consist of equiaxed grains and large fraction of high-angle grain boundaries.
3. The substructured grains were unstable, which could be transformed into the recrystallized grains easily after MEM process.

Moreover, the specimens processed by MEM, MEE, MEM-MEM, and MEE-MEE were further analyzed through TEM, and the conclusions are made below.

1. The dislocation cells were formed by the slip and the accumulation of dislocations, which could be transformed into sub-grains with the increase of the strain.
2. With the repetitive process of CECC, the uniformity of microscopic distribution of secondary phases markedly improved.
3. The secondary phase of Mg_2_Si function as nucleation substrate and the refining performance of 6061 aluminum alloy. Moreover, the secondary phase of Mg_2_Si were pinned at the grain boundaries, preventing the refined grains from growing up again.

The dislocation proliferation and secondary phases will both lead to grain refinement. The results, accompanied by the enhanced mechanical properties of UFG, allow the prediction that specimens may have an ultrafine-grained structure produced by the CECC routes of MEM and may yield attractive products when subjected to further processing. 

## Figures and Tables

**Table 1:** Chemical composition (in wt.%) of 6061 aluminum alloy.

Si	Fe	Cu	Mn	Mg	Cr	Zn	Ti	Al
0.45	0.21	0.18	0.15	0.95	0.08	0.25	0.15	Bal.

**Table 2:** Mechanical properties of 6061 aluminum alloy processed by different SPD (Severe plastic deformation) methods.

SPD Methods	Ultimate Strength/(Pa)	Elongation/(%)	Microhardness/(Hv)
Undeformed and annealed	128	17.3	56.4
3-pass of ECAP	270	10.9	92.5
3-pass of MAC	276	10.2	88.1
MEM	320	12.8	115.2
MEE	283	11.1	101.5

**Table 3:** The average grain size of Al-6061 alloy (as received state, annealed state, and after the CECC process).

Treatment Scheme	Non-Treatment	Annealed and Undeformed	MEM	MEE	3-Pass of ECAP	3-Pass of MAC
Average grain size/(μm)	21	28	1.2	1.6	2.1	2.8

**Table 4:** The recrystallization statistics of 6061 aluminum alloys processed by different routes of CECC.

CECC Routes	MEM	MEE	3-Pass of ECAP	3-Pass of MAC
Recrystallized/(%)	25.8	20	13.6	5.5
Substructured/(%)	50	67	73.4	38.5
Deformed/(%)	24.2	13	13	56
